# The neutrophil-to-lymphocyte and monocyte-to-lymphocyte ratios are independently associated with neurological disability and brain atrophy in multiple sclerosis

**DOI:** 10.1186/s12883-019-1245-2

**Published:** 2019-02-12

**Authors:** Christopher C. Hemond, Bonnie I. Glanz, Rohit Bakshi, Tanuja Chitnis, Brian C. Healy

**Affiliations:** 1 Department of Neurology, University of Massachusetts Medical Center, 55 Lake Ave North, Worcester, MA 01655 USA; 2 Department of Neurology, Harvard Medical School, Boston, MA USA; 3 Partners Multiple Sclerosis Center, Ann Romney Center for Neurologic Diseases, Brigham & Women’s Hospital, Boston, MA USA; 4 Department of Radiology, Harvard Medical School, Boston, MA USA

**Keywords:** Multiple sclerosis, Neutrophil-to-Lympocyte ratio, Monocyte-to-Lympocyte ratio, Brain atrophy, MRI, Patient-reported outcomes

## Abstract

**Background:**

Serum hematological indices such as the neutrophil-lymphocyte ratio (NLR) or monocyte-lymphocyte ratio (MLR) have been used as biomarkers of pathogenic inflammation and prognostication in multiple areas of medicine; recent evidence shows correlation with psychological parameters as well.

Objectives/Aims: To characterize clinical, neuroimaging, and psycho-neuro-immunological associations with NLR and MLR in persons with multiple sclerosis (MS).

**Methods:**

We identified a large cohort of clinically well-defined patients from our longitudinal database that included MS-related outcomes, disease-modifying therapy, patient-reported outcome (PRO) measures, and quantified cerebral MRI at 1.5 T. We queried hospital records for complete blood counts within 2 months of each clinic visit and excluded those obtained during clinical relapses. Four hundred eighty-three patients, with a mean of 3 longitudinal observations each, were identified who met these criteria. Initial analyses assessed the association between NLR and MLR as the outcomes, and psychological and demographic predictors in univariable and multivariable models controlling for age, gender and treatment. The second set of analyses assessed the association between clinical and MRI outcomes including whole brain atrophy and T2-hyperintense lesion volume, with NLR and MLR as predictors in univariable and multivariable models. All analyses used a mixed effects linear or logistic regression model with repeated measures.

**Results:**

Unadjusted analyses demonstrated significant associations between higher (log-transformed) NLR (but not MLR) and PRO measures including increasing depression (*p* = 0.01), fatigue (*p* < 0.01), and decreased physical quality of life (p < 0.01). Higher NLR and MLR strongly predicted increased MS-related disability as assessed by the Expanded Disability Status Scale, independent of all demographic, clinical, treatment-related, and psychosocial variables (*p* < 0.001). Lastly, higher NLR and MLR significantly discriminated progressive from relapsing status (*p* ≤ 0.01 for both), and higher MLR correlated with increased whole-brain atrophy (*p* < 0.05) but not T2 hyperintense lesion volume (*p* > 0.05) even after controlling for all clinical and demographic covariates. Sensitivity analyses using a subset of untreated patients (*N* = 146) corroborated these results.

**Conclusions:**

Elevated NLR and MLR may represent hematopoetic bias toward increased production and pro-inflammatory priming of the myeloid innate immune system (numerator) in conjunction with dysregulated adaptive immune processes (denominator), and consequently reflect a complementary and independent marker for severity of MS-related neurological disability and MRI outcomes.

**Electronic supplementary material:**

The online version of this article (10.1186/s12883-019-1245-2) contains supplementary material, which is available to authorized users.

## Background

### Introduction

Multiple sclerosis (MS) is a progressive neurodegenerative condition of presumed autoimmune etiology typically characterized by recurrent episodes of inflammatory demyelination and accelerated tissue destruction of the central nervous system. The etiology of the disease is thought to principally involve the peripheral activation of Th1 lymphocytes which traffic to the central nervous system and react with myelin autoantigens. Following activation, these T-cells recruit a diverse set of myeloid cells including monocytes/macrophages to promote and execute an inflammatory reaction, frequently causing axonal transection and irreversible focal central nervous system (CNS) injury [1]. In addition to monocytes, neutrophils have also recently been implicated to play a role in augmentation of the inflammatory response in MS [2].

The neutrophil-lymphocyte ratio (NLR) and monocyte-lymphocyte ratio (MLR) are inexpensive and readily available components of the standard complete blood count (CBC); they are increasingly recognized as clinically-relevant biomarkers of pathological inflammation across multiple areas of medicine including oncology [3, 4], cardiovascular disease [5], and autoimmune conditions [6]. In neurology, NLR improves outcome prediction in stroke [7, 8], and is elevated compared to healthy controls in degenerative conditions like Alzheimer’s [9, 10] and Parkinson’s [11] disease.

The NLR and MLR have also been linked to psychological parameters. The NLR has a well-established short-term response to both endogenous and exogenous corticosteroids [12] and links to perceived stress in humans [13]. A recent meta-analysis concluded that there is an elevated NLR in several mood disorders including depression [14]. In a population of patients with stable coronary artery disease, Serfozo and colleagues showed a strong independent correlation (after controlling for age, body mass index and sex) between the MLR and depression symptom severity score, as well as other inflammatory markers (IL-6, C-Reactive Protein), and neuroendocrine-sympathetic activity as measured by chromogranin A [15]. It has been proposed that psychosocial stress and related chronic psychiatric diseases (depression, anxiety) may bias the hemopoetic differentiation process toward both (1) increased production of monocytes and granulocytes and (2) enhance myeloid pro-inflammatory ‘priming’ and trafficking, both via bidirectional interactions between CNS sympathic/neuroendocrine systems and lymphoid tissue [16–18]. Irrespective of psychosocial factors however, both neutrophils and monocytes have been observed to be ‘primed’ for pro-inflammatory activity in persons with multiple sclerosis, thought to be related to the chronic systemic inflammatory environment of this disease [2, 19].

There is limited prior literature regarding the NLR in MS; Demirci and colleagues were the first to explore this area and found that an elevated NLR could discriminate MS from healthy controls, was related to neurological disability scores, and was further increased in flares compared to remission [20]. Several subsequent studies have confirmed similar findings [13, 20, 21], although one study could not replicate associations with disability scores [22]. None of these studies carefully examined the role of disease-modifying therapies however; nor did they look at MRI-related disease outcomes such as cerebral atrophy or quantified T2-hyperintense lesion volume. The role of MLR has not been studied in MS to our knowledge. This work has been previously published in abstract format [23].

### Objectives/aims

Here we hypothesize that the NLR and MLR are psycho-neuro-immunological markers; modulated in part by factors such as stress, depression, and social isolation/support. Furthermore, we hypothesize that the NLR and MLR play a role in MS-related disability by contributing to (or reflecting) a pro-inflammatory state. To this end, our first aim was to establish whether a relationship exists between the NLR/MLR and demographic variables including age and sex, as well as MS-related clinical phenotypes, disease duration, and disease-modifying therapies. Second, we sought to better understand associations between the NLR/MLR and patient-reported psychological outcomes including mental health, social support, depression, and quality of life. Third, we aimed to explore associations with objective markers of neuroinflammation and degeneration as reflected by neurological disability and MRI neuroimaging parameters relevant in MS, including whole brain atrophy and total burden of cerebral T2-hyperintense lesions.

## Methods

### Subjects

This is a retrospective analysis of prospectively-collected data in a cohort of multiple sclerosis subjects who participated in the Comprehensive Longitudinal Investigation of Multiple Sclerosis at the Brigham and Women’s Hospital, Partners MS Center (CLIMB). CLIMB is an ongoing, prospective observational cohort study, started in 2000 [24]. General inclusion criteria to participate include: age ≥ 18 years and a diagnosis of relapsing-spectrum or progressive MS syndrome based on revised McDonald Criteria [25]. MS participants are examined at a minimum of every 12 months by a neurologist with subspecialty MS training; demographic and clinical data are regularly collected as part of this study. Additionally, a subset of patients consented to complete patient-reported outcome measures (PRO) as detailed below. Additional inclusion criteria for this specific study were: age < 65, and a CBC with differential within 60 days of their patient-reported outcome assessment. Exclusion criteria included: CBC-d obtained on the same day or the following 1 month after reported clinical flare, or multiple CBCs within 4 days of each other which were thought to have a high probability of being obtained during times of infection or inpatient hospitalization. In total, 483 patients were included in the analysis, with a mean of 3.0 observations each (range: 1–10) for a total of 1433 observations. Subject demographics, clinical characteristics, patient self-reported outcome measures, and neuroimaging data are summarized in Table 1.

**Table 1:** Clinical and demographic characteristics, neuroimaging, and patient-reported outcomes

Variable	Median (IQR) or N (%)
N	483
Age (years)	44.5 (36, 51.9)
Disease Duration (years)	8.8 (5.3, 16)
Neutrophil-lymphocyte ratio	2.1 (1.54, 2.9)
Monocyte-lymphocyte ratio	0.2 (0.15, 0.28)
Expanded Disability Status Scale	2 (1, 3)
Gender	
• Female	347 (71.8%)
• Male	136 (28.2%)
Smoking History^a^	
• No	233 (54.2%)
• Yes	197 (45.8%)
Body mass index (BMI)^a^	26.2 (22.8, 31.1)
Multiple sclerosis disease category	
• Clinically isolated syndrome	15 (3.1%)
• Relapsing-remitting	370 (76.6%)
• Secondary progressive	65 (13.5%)
• Primary progressive	33 (6.8%)
Disease modifying therapy	
• Untreated	110 (22.8%)
• Interferon-β	216 (44.7%)
• Cyclophosphamide	14 (2.9%)
• Fingolimod	8 (1.7%)
• Glatiramer Acetate	51 (10.6%)
• Mycophenolate Mofetil	33 (6.8%)
• Natalizumab	37 (7.7%)
• Other	14 (2.9%)
Neuroimaging and patient-reported outcome measures	Median (IQR); N
Brain parenchymal fraction	0.85 (0.81, 0.88); 334
Cerebral T2-hyperintense lesion volume (mL)	1.57 (1.27, 2.03); 334
Center for Epidemiological Studies Depression Scale	29 (24, 36); 479
Modified Fatigue Inventory Scale	29 (14, 41); 425
Modified Social Support Scale	85.4 (71.1, 98.4); 477
SF-36 Physical composite	46.6 (37.9, 54.3); 471
SF-36 Mental composite	50.9 (42.5, 56.4); 471

Legend: “Other” DMT includes combinations of or more of the following: methotrexate, azathioprine, pulsed glucocorticoids, anti-IL2 agents, rituximab, natalizumab, interferons, glatiramer acetate, cyclophosphamide. Cyclophosphamide, methotrexate and mycophenolate mofetil were occasionally combined with an interferon or glatiramer acetate; otherwise medications are listed as monotherapy. Median and interquartile ranges use data from patient’s most recent available clinical visit. ^a^Smoking history (*N* = 430), BMI (*N* = 402)

### Consent to participate

This study was reviewed and approved by the Partners Human Research Committee ethics board at the Brigham and Women’s Hospital (IRB Protocol # 1999-P-010435). All research subjects provided informed written consent.

### Patient-reported measures

Four patient-reported psychological questionnaires are administered to a subset of participants in the CLIMB study as follows. The 20-item Center for Epidemiology Depression scale (CES-D) [26] is a validated scale that focuses on the cognitive and affective rather than the somatic components of depression. Scores range from 20 to 80 and a cutoff of ≥36 is considered to be in the depressed range. The 21-item Modified Fatigue Inventory Scale (MFIS) [27] assesses physical, cognitive, and psychosocial fatigue with a score range from 0 to 84; a cutoff of ≥38 represents clinically meaningful fatigue. The SF-36 [28] is an inventory of mental, physical, and social measures of life satisfaction and fitness that assesses eight health concepts and is summarized in two composite scores of physical and mental quality of life. The 18-item Medical Outcomes Modified Study Social Support Survey (MSSS) [29] is an assessment of patient’s perceived social support with four subscales and raw scores ranging from 18 to 90. PROs were administered annually at study inception (2000) until 2011 when they began being collected biennially. The exception is the MFIS, which was added on 6/23/2006.

### Complete blood counts

Automated complete blood counts with differential were obtained via an electronic query of hospital records. Personal communication with hematology, as well as reference test numbers, confirmed the use of two automated hematological analysis machines during the data collection period: Siemens ADVIA 2120 was used prior to 10/29/2011 and a SYSMEX XE 5000 was used after 10/29/2011. The vast majority of the data were from the ADVIA 2120, so measurements from the SYSMEX XE were excluded from the study to reduce variability (see Discussion).

### Neuroimaging

Brain MRI scans were acquired at 1.5 T from several Signa GE scanners with similar dual-echo spin-echo acquisition parameters: TR = 2800–3000 msec, TE1/TE2 = 30/80 msec, slice thickness = 3 mm (gapless), pixel size = 0.93 × 0.93 mm. Brain parenchymal fraction (BPF) and cerebral T2-hyperintense lesion volumes (T2LV) were obtained from automated template-driven segmentation [30] with additional expert manual correction as required. Brain parenchymal fraction was defined as the summation of cerebral gray and white matter divided by volume of the intracranial cavity to yield a self-normalized fraction. T2LV was quantified a summated single volume measurement.

### Statistical analysis

Statistical analysis of the demographic, clinical, and laboratory variables was performed using the R (http://www.R-project.org) or Stata v14.0 (www.stata.com) software. Normality of variables was assessed by a combination of skew/kurtosis metrics, visual inspection of the histogram distribution and the Shapiro-Wilk normality test. NLR/MLR did not meet normality criteria and was therefore natural log (base *e*) transformed. Most other variables were skewed so medians and interquartile ranges are reported as opposed to means and standard deviations. In our first set of analyses, we assessed which demographic, treatment, and psychosocial variables were associated with the concurrent natural log-transformed NLR or MLR. For this analysis, we accounted for repeated observations on subjects using a linear mixed effects model with a subject-specific random intercept. Each potential predictor was included in the model separately in the univariable analysis. Further, a multivariable model including demographic factors, smoking history and specific disease-modifying therapy (DMT) use together was also fit to estimate the independent association of each feature accounting for these covariates. In addition to the analysis including all measurements per subject, a separate univariate analysis including only the most recent time point was performed as well, and included as Additional file 1. In the second set of analyses, we assessed whether NLR or MLR was associated with clinical and radiologic measures of disease severity using a linear mixed effects model with a subject specific random intercept. In this model, the EDSS, BPF, cubic-transformed T2 lesion volume, and clinical disease category were the outcome measures. We assessed the univariable association including only NLR or MLR, and we also fit 2 different multivariable models. Model 1 included demographic and treatment predictors, whereas model 2 included demographic, treatment and psychosocial predictors to assess the independent contribution of NLR and MLR on the outcomes. Lastly, we assessed whether baseline NLR or MLR could predict subsequent (longitudinal) worsening of a patient’s MS-related disability using EDSS as the outcome variable in a linear mixed effects model with repeated measures. In this model the predictor variables included time elapsed (days) since baseline measurement, the natural log-transformed MLR or NLR, and the interaction between the two maintained as continuous variables.

## Results

### Clinical, demographic, and self-reported outcomes associated with the NLR and MLR

Univariable and multivariable associations between demographic and psychosocial measures with NLR are provided in Table 2, and MLR in Table 3. In both univariable and multivariable comparisons controlling for age, gender, smoking history, and DMT, NLR was significantly higher for subjects taking cyclophosphamide and fingolimod, and significantly lower for subjects taking interferons and natalizumab. Increased NLR was also significantly associated with higher depression (CES-D) scores, higher fatigue scores (MFIS), and lower physical quality of life (SF-36), but not social support as assessed by the MSSS, or BMI (both *p* > 0.05). Uni- and multi-variable analyses with MLR as the outcome showed similar significant associations with the same DMTs as NLR except directionality reversal for interferons; additionally, higher MLR significantly correlates with older age, longer disease duration (univariable *p* < 0.01; multivariable *p* = 0.052) and male gender, but not with any patient-reported outcome measures.

**Table 2:** Clinical, demographic, and patient self-reported associations with the (natural log-transformed) Neutrophil-Lymphocyte Ratio

	Univariable			Multivariable		
	*β*	95% CI	*p*	*β*	95% CI	*p*
Age (yrs)	0.023	− 0.011, 0.056	0.188	0.021	− 0.010, 0.053	0.189
Disease Duration (yrs)	0.009	−0.023, 0.040	0.591	−0.013	− 0.048, 0.022	0.477
Gender						
• Female	–	–	–	–	–	–
• Male	−0.003	−0.083, 0.077	0.942	−0.012	−0.086, 0.062	0.750
Smoking History						
• No	–	–	–	–	–	–
• Yes	0.001	−0.076, 0.078	0.979	0.018	− 0.054, 0.090	0.621
BMI	−0.008	− 0.045, 0.029	0.666	0.002	−0.033, 0.037	0.910
DMT						
• Untreated	–	–	–	–	–	–
• Interferon-β	−0.166	− 0.236, − 0.096	**< 0.001**	− 0.163	−0.233, − 0.093	**< 0.001**
• Cyclophosphamide	0.268	0.147, 0.389	**< 0.001**	0.274	0.152, 0.395	**< 0.001**
• Fingolimod	1.044	0.767, 1.320	**< 0.001**	1.047	0.770, 1.323	**< 0.001**
• Glatiramer Acetate	−0.082	−0.187, 0.024	0.131	−0.079	−0.185, 0.027	0.142
• Mycophenolate	−0.072	−0.181, 0.037	0.197	−0.075	− 0.184, 0.035	0.181
• Natalizumab	−0.529	−0.647, − 0.411	**< 0.001**	−0.526	− 0.644, − 0.408	**< 0.001**
• Other	− 0.009	−0.141, 0.124	0.900	−0.012	− 0.144, 0.121	0.862
CES-D	0.034	0.008, 0.061	**0.012**	0.030	0.004, 0.055	**0.022**
MFIS	0.045	0.012, 0.078	**0.008**	0.035	0.004, 0.066	**0.029**
MSSS	−0.019	−0.047, 0.010	0.203	−0.021	−0.048, 0.006	0.121
SF-36 Physical QOL	−0.055	−0.086, − 0.024	**< 0.001**	−0.037	− 0.067, − 0.007	**0.015**
SF-36 Mental QOL	− 0.024	−0.050, 0.003	0.086	−0.024	− 0.049, 0.002	0.066

Legend: *CES-D* Center for epidemiology scale for depression, *MFIS* Modified fatigue impact scale, *MSSS* Modified social support survey, *QOL* Quality of life, *BMI* Body mass index, *DMT* Disease modifying therapy. Mixed linear regression models use data from all patient visits (repeated measures), with multivariable model including the following covariates: age, gender, smoking history, and DMT use. Continuous predictor variables are Z-transformed such that one unit increase represents one standard deviation change. Bolded values represent *p* < 0.05

**Table 3:** Clinical, demographic, and patient self-reported associations with the (natural log-transformed) Monocyte-lymphocyte Ratio

	Univariable			Multivariable		
	*β*	95% CI	*p*	*β*	95% CI	*p*
Age (yrs)	0.051	0.019, 0.082	**0.002**	0.057	0.028, 0.086	**< 0.001**
Disease Duration (yrs)	0.043	0.013, 0.072	**0.004**	0.033	0.000, 0.065	**0.049**
Gender						
• Female	–	–	–	–	–	–
• Male	0.163	0.090, 0.237	**< 0.001**	0.160	0.092, 0.229	**< 0.001**
Smoking History						
• No	–	–	–	–	–	–
• Yes	−0.002	−0.074, 0.070	0.958	0.003	−0.063, 0.069	0.937
BMI	−0.031	−0.066, 0.003	0.074	−0.024	− 0.056, 0.007	0.132
DMT						
• Untreated	–	–	–	–	–	–
• Interferon-β	0.068	0.004, 0.132	**0.038**	0.077	0.013, 0.140	**0.018**
• Cyclophosphamide	0.296	0.187, 0.406	**< 0.001**	0.310	0.201, 0.418	**< 0.001**
• Fingolimod	1.219	0.970, 1.469	**< 0.001**	1.207	0.959, 1.454	**< 0.001**
• Glatiramer Acetate	−0.045	−0.142, 0.052	0.360	−0.047	−0.142, 0.049	0.339
• Mycophenolate	0.062	−0.037, 0.162	0.219	0.063	−0.035, 0.162	0.206
• Natalizumab	−0.378	−0.486, − 0.270	**< 0.001**	−0.374	− 0.48, − 0.267	**< 0.001**
• Other	0.057	− 0.063, 0.178	0.350	0.048	−0.071, 0.168	0.426
CES-D	0.010	−0.015, 0.034	0.437	0.007	−0.016, 0.030	0.534
MFIS	0.008	−0.022, 0.038	0.604	0.005	−0.022, 0.033	0.704
MSSS	0.007	−0.019, 0.033	0.604	0.006	−0.018, 0.031	0.611
SF-36 Physical QOL	−0.017	−0.045, 0.012	0.254	−0.011	− 0.038, 0.016	0.441
SF-36 Mental QOL	−0.005	−0.030, 0.019	0.676	−0.005	− 0.027, 0.018	0.678

Legend: *CES-D* Center for epidemiology scale for depression, *MFIS* Modified fatigue impact scale, *MSSS* Modified social support survey, *QOL* Quality of life, *BMI* Body mass index, *DMT* Disease modifying therapy. Mixed linear regression models use data from all patient visits (repeated measures), with multivariable model including the following covariates: age, gender, smoking history, and DMT use. Continuous predictor variables are Z-transformed such that one unit increase represents one standard deviation change. Bolded values represent *p* < 0.05

### NLR/MLR are associated with MS-related disability and neuroimaging outcomes

We then examined disease category (progressive vs. relapsing), physical disability status (EDSS score), and neuroimaging outcome measures (BPF and T2LV) with results summarized in Tables 4 and 5. In all models, higher NLR and MLR were both robustly associated with increased EDSS scores; effect sizes were slightly larger using MLR (*β* ~ 0.26) compared to NLR (*β* ~ 0.21). Higher NLR and MLR both significantly discriminated progressive from relapsing forms of MS in univariable and basic multivariable analyses, although only NLR significantly added to the complex multivariable analysis (model 2) using age, disease duration, gender, smoking history, DMT, depression, social support, and quality of life measures as covariates (*β* = 0.45, *p =* 0.003). With BPF as an outcome, higher MLR, but not NLR, was significantly associated with greater whole brain atrophy in all statistical models (*p* < 0.05). Lastly, neither NLR or MLR were good correlates of T2 lesion volume, although univariable comparison with MLR was borderline (*p* = 0.055). We did not include MFIS in the multivariable model due to the decreased sample size (*N* = 424); when we did include it, results (model #2) were similar. Given the strong associations with DMT (Table 2), we also performed sensitivity analyses using untreated patients only (*N* = 146), and found 3–7 times increase in effect size for associations with EDSS and disease category (relapsing vs. progressive MS) in all linear models: see Additional file 1 for full information.

**Table 4:** Natural log-transformed neutrophil-lymphocyte ratio as a predictor of objective MS disease outcomes

Outcome	Univariable			Multivariable Model 1			Multivariable Model 2		
	*β*	95% CI	*p*	*β*	95% CI	*p*	*β*	95% CI	*p*
EDSS	0.208	0.078, 0.338	**0.002**	0.238	0.106, 0.369	**< 0.001**	0.217	0.082, 0.351	**0.002**
RRMS v PMS	0.219	0.093, 0.344	**0.001**	0.599	0.350, 0.849	**< 0.001**	0.452	0.157, 0.747	**0.003**
BPF	−0.001	−0.004, 0.002	0.610	−0.001	− 0.004, 0.001	0.243	− 0.002	−0.004, 0.001	0.156
T2LV	−0.001	−0.032, 0.031	0.970	0.002	−0.031, 0.035	0.912	0.006	−0.029, 0.041	0.753

Legend: *EDSS* Expanded disability status scale, *RRMS* Relapsing-remitting multiple sclerosis, *PMS* Progressive multiple sclerosis, *BPF* Brain parenchymal fraction, *T2LV* Cerebral T2-hyperintense lesion volume. All statistical models are linear mixed effects regressions. Multivariable model #1 includes the following covariates: age, disease duration, gender, and specific DMT use. Model #2 includes the covariates: age, disease duration, gender, smoking history, specific DMT use, CES-D score, MSSS score, SF-36 physical and mental composite scores

**Table 5:** Natural log-transformed monocyte-lymphocyte ratio as a predictor of objective MS disease outcomes

Outcome	Univariable			Multivariable Model 1			Multivariable Model 2		
	*β*	95% CI	*p*	*β*	95% CI	*p*	*β*	95% CI	*p*
EDSS	0.269	0.125, 0.413	**< 0.001**	0.256	0.108, 0.404	**0.001**	0.249	0.096, 0.401	**0.001**
RRMS v PMS	0.198	0.048, 0.347	**0.010**	0.407	0.158, 0.655	**0.001**	0.292	−0.062, 0.647	0.106
BPF	−0.006	−0.010, − 0.003	**< 0.001**	−0.003	− 0.006, 0.000	**0.043**	− 0.003	−0.006, 0.000	**0.040**
T2LV	0.035	−0.001, 0.071	0.055	0.019	−0.019, 0.057	0.329	0.031	−0.009, 0.071	0.124

Legend: *EDSS* Expanded disability status scale, *RRMS* Relapsing-remitting multiple sclerosis, *PMS* Progressive multiple sclerosis, *BPF* Brain parenchymal fraction, *T2LV* Cerebral T2-hyperintense lesion volume. All statistical models are linear mixed effects regressions. Multivariable model #1 includes the following covariates: age, disease duration, gender, and specific DMT use. Model #2 includes the covariates: age, disease duration, gender, smoking history, specific DMT use, CES-D score, MSSS score, SF-36 physical and mental composite scores

### NLR/MLR does not predict longitudinal worsening of disability

Using only subjects with longitudinal data (*N* = 329, 1279 total observations), we tested whether baseline (first measurement) NLR or MLR could predict subsequent worsening of EDSS using the interaction term: (time elapsed)*(ln)M/NLR. This interaction was neither significant in all subjects (NLR *p* = 0.15; MLR *p* = 0.22), nor a subset of early relapsing patients (disease duration < 5 years, *N* = 144, NLR *p* = 0.77; MLR *p* = 0.49), nor a cohort of early relapsing untreated patients (*N* = 27, NLR *p* = 0.08; MLR *p* = 0.64).

## Discussion

The field of multiple sclerosis continues to lack a comprehensive serum biomarker for the diagnosis or monitoring of disease activity. Here we present evidence that the common hematologic index ratios of neutrophil-to-lymphocyte (NLR) and monocyte-to-lymphocyte (MLR) are both robustly associated with neurological disability scores in MS, independent of all demographic and clinical variables. Additionally, the MLR is associated with the MRI outcome of whole brain atrophy (BPF), but not inflammatory burden of T2-hyperintense cerebral lesions (T2LV). A higher NLR (and MLR to a lesser extent) associate strongly with a diagnosis of progressive disease in MS. NLR, but not MLR, is additionally associated with self-reported patient outcomes including depression, fatigue, and quality of life, and thus represents a psycho-neuro-immunological marker. Strengths of this study include the large and well-characterized sample of MS patients, including self-reported psychological outcome measures, specific DMT use, and quantified neuroimaging pathology. Together this dataset allows robust statistical modeling and higher confidence in the results; to our knowledge, this is the largest report of NLR in multiple sclerosis, the first to address confounding with DMT use, and include characterization of neuroimaging findings; this is the first reporting of the MLR in MS as well.

Our findings generally corroborate the limited prior literature in this specific area, which consists of four case-control, and one retrospective cohort study. All case-control studies found significantly elevated NLR in MS (or optic neuritis) compared to controls [13, 20, 22]. All groups found a mean NLR > 2.1 in MS compared to healthy controls NLR < 2.1, however we urge caution before directly comparing trials since we found the CBC analysis machine to be a significant source of variability in our data and therefore excluded the use of the SYSMEX XE machine (results not shown). This methodological observation limits inter-study comparisons, and carries implications for the role of NLR/MLR as potential biomarkers. Several of these prior studies also found a higher NLR during flares compared to remission. We did not attempt to replicate those results in our data due to the uncertain timing of (likely) intravenous glucocorticoid administration during flares; this medication would significantly elevate the NLR through demargination of neutrophils and we therefore excluded all CBC measurements during flares.

Three studies have looked at the role of NLR as a correlate of disability in MS; two of these demonstrated a strong univariable [21] or multivariable [20] association, discriminating EDSS scores of 5 and 3, respectively; whereas one group found no significant value of NLR to predict EDSS> 4 in a cross-sectional multivariable model including age, gender, and disease duration [22]; this latter study also found no association with clinical phenotype (relapsing vs progressive MS). Here, our results support a strong association between higher NLR/MLR and worse disability scores in robust multivariable models; these measures are similarly able to significantly discriminate progressive from relapsing patients. However, despite robust correlations with disability and substantial prior literature suggesting a role for the NLR in prognostication, a limited longitudinal analysis did not reveal a role for NLR or MLR in predicting future disability worsening. Our retrospective analysis was not properly designed to answer this question however, so this data should be interpreted cautiously and further investigation is warranted.

The disease-modifying treatments used in the contemporary treatment of MS are, by design, highly immunologically active and clearly affect the NLR and MLR. Most DMTs except glatiramer acetate have well-established (and possibly nonlinear) effects on the numbers of circulating leukocytes; most relevant here are fingolimod (lymphopenia/monocytosis) [31], natalizumab (lymphocytosis) [32], cyclophosphamide (lymphopenia), and interferon-beta (lymphopenia/neutropenia) [33], effects that are confirmed from our data (Table 2). Overall these medications clearly limit the utility of the NLR/MLR as cross-sectional bedside translational markers- a challenge with any biomarker in MS. However, these effects may be accounted for as covariates as we did here; sensitivity analyses using only untreated patients typically made the associations between NLR/MLR and MS outcomes such as EDSS even more robust (see Additional file 1). Ours is the only study in the literature to statistically account for the effects of DMT, and our results therefore suggest a possible role in clinical translation, especially given the low cost and widespread accessibility of this marker.

Lastly, Al-Hussain et al. [13] focused their paper on the significant associations between NLR and psychological stress (*p* < 0.001). Whereas they did not find significant associations with depression or anxiety, our analysis demonstrated a small but highly significant association between NLR and depression after controlling for age, gender, smoking history and DMT, similar to results of a recent meta-analysis of NLR in mood disorders [14]. We could not replicate prior findings of an association between MLR and social support [34]. These psychoneuroimmunological associations should be further explored as potential avenues of treatment that interact with neuroinflammation.

More work is needed to better elucidate the mechanistic immune feedback loops that presumably underlie the origin of absolute myeloid and lymphocyte counts in the circulating blood compartment; especially regarding the (temporal) dynamics involved in relative rates of leukocyte production, sequestration, egress, and survival dynamics which are all integrated into these markers. In a general sense, the numerator represents nonspecific activation of the innate (myeloid) immune system, and the denominator reflects suppression of more adaptive processes. Higher circulating monocytes (MLR) were associated with greater whole brain atrophy and also with male gender, the latter being a well-known risk for more rapid disability accumulation [35]. The observation that the NLR and MLR are significantly associated with neuroinflammation-related disability, disease category, and brain atrophy outcomes, also reflects current thinking in the multiple sclerosis literature regarding a transition from adaptive to (upregulated) innate immune processes during the conversion phase from relapsing to progressive clinical disease [36, 37]. This clinical transition is also marked by a plateau effect of T2-hyperintense lesions [38], a finding likely reflected in our data by the lack of association between NLR/MLR and T2LV.

Multiple limitations exist in this study, perhaps most importantly that our results are of an associative nature only, and thus no conclusions should be drawn regarding causality. Complex bidirectional relationships exist regarding the connection between psychosocial (patient-reported) factors, immune function, and disease outcomes; our study only provides a limited insight into how these factors interact. Additionally this study is limited by its retrospective nature that is susceptible to selection and information biases; these include unclear motivations for ordering CBCs among untreated patients, or those on glatiramer acetate in whom hematological monitoring is not required. Patients in the untreated group may still be affected by prior drug washout. Some of our cohort is missing neuroimaging outcome data or patient-reported outcomes, which reduces power in certain analyses and may bias results. Further, we lack insight into subjects’ medical comorbidities or other medication use which may affect the NLR/MLR. Lastly, the CBC-d only provides a gross metric of immune function and is limited in resolving lympocyte subsets such as Th1/Th17 that are important in MS pathogenesis [39, 40].

## Conclusions

As a readily available and widespread diagnostic and monitoring tool, the NLR and MLR may represent translational metrics that can provide independent and complementary cross-sectional information to a patient’s inflammatory disease status in multiple sclerosis.

## Additional file

Additional file 1: **Tables S1**-**S4** Sensitivity analyses using cross-sectional data only, using a single time point (subjects’ last clinic visit), and are otherwise analogous to Tables 2, 3, 4 and 5 in the manuscript. **Tables S5.** and **S6.** Sensitivity analyses using a subset of patients who were untreated (not on a disease-modifying therapy) at the time of their clinical visit. (DOCX 53 kb)

## Abbreviations

BMI: Body mass index
BPF: Brain parenchymal fraction
CBC: Complete blood count
CBC-d: Complete blood count with differential
CES-D: Center for Epidemiology Depression scale
CLIMB: Comprehensive Longitudinal Investigation of Multiple sclerosis in Boston
CNS: Central nervous system
DMT: Disease-modifying therapy
EDSS: Expanded disability status scale
MFIS: Modified Fatigue Inventory Scale
MLR: Monocyte-to-lymphocyte ratio
MRI: Magnetic resonance imaging
MS: Multiple sclerosis
MSSS: Modified Study Social Support Survey
NLR: Neutrophil-to-lymphocyte ratio
PRO: Patient-reported outcome
T2LV: Cerebral T2-hyperintense lesion volumes
