# Supplementary material for: The neutrophil-to-lymphocyte and monocyte-to-lymphocyte ratios are independently associated with neurological disability and brain atrophy in multiple sclerosis
Source: BMC Neurol. 2019 Feb 12;19:23. doi: 10.1186/s12883-019-1245-2 (PMC6371437; doi:10.1186/s12883-019-1245-2)
Supplement: Supplementary file 1 — Tables S1-S4 Sensitivity analyses using cross-sectional data only, using a single time point (subjects’ last clinic visit), and are otherwise analogous to Tables 2, 3, 4 and 5 in the manuscript. Tables S5. and S6. Sensitivity analyses using a subset of patients who were untreated (not on a disease-modifying therapy) at the time of their clinical visit. (DOCX 53 kb) [file 12883_2019_1245_MOESM1_ESM.docx]

**Additional file 1**

Tables S1-S4: Sensitivity analyses using cross-sectional data only using a single time point (subjects’ last clinic visit), and are otherwise analogous to tables 2-5 in the manuscript.

SI Tables 5 and 6: Sensitivity analyses using a subset of patients who were untreated (not on a disease-modifying therapy) at the time of their clinical visit.

| Table S1: Predictors of natural log-transformed NLR in univariate analysis (last subject visit) | | | | | | |
| --- | --- | --- | --- | --- | --- | --- |
| Variable | *Univariable* | | | *Multivariable* | | |
|  | *β* | 95% CI | *p* | *β* | 95% CI | *p* |
| Age (yrs) | 0.056 | 0.014, 0.099 | 0.010 | 0.046 | 0.003, 0.088 | 0.035 |
| Disease Duration (yrs) | 0.047 | 0.006, 0.088 | 0.026 | -0.016 | -0.063, 0.031 | 0.499 |
| Gender |  |  |  |  |  |  |
| - Female | - | - | - | - | - | - |
| - Male | 0.018 | -0.080, 0.117 | 0.714 | 0.020 | -0.077, 0.116 | 0.689 |
| Smoking History |  |  |  |  |  |  |
| - No/Unknown | - | - | - | - | - | - |
| - Yes | 0.033 | -0.062, 0.129 | 0.494 | 0.047 | -0.040, 0.134 | 0.292 |
| BMI | 0.000 | -0.008, 0.008 | 0.931 | -0.002 | -0.049, 0.044 | 0.924 |
| DMT |  |  |  |  |  |  |
| - Untreated | - | - | - | - | - | - |
| - Interferon-β | -0.205 | -0.307, -0.103 | <0.001 | -0.171 | -0.283, -0.059 | 0.003 |
| - Cyclophosphamide | 0.429 | 0.181, 0.676 | 0.001 | 0.473 | 0.202, 0.745 | 0.001 |
| - Fingolimod | 1.043 | 0.723, 1.362 | <0.001 | 1.119 | 0.770, 1.467 | <0.001 |
| - Glatiramer Acetate | -0.129 | -0.277, 0.019 | 0.087 | -0.104 | -0.262, 0.053 | 0.193 |
| - Mycophenolate | -0.040 | -0.213, 0.133 | 0.649 | -0.044 | -0.226, 0.137 | 0.631 |
| - Natalizumab | -0.501 | -0.666, -0.335 | <0.001 | -0.477 | -0.650, -0.303 | <0.001 |
| - Other | 0.139 | -0.109, 0.386 | 0.272 | 0.164 | -0.098, 0.426 | 0.220 |
| CES-D | 0.028 | -0.016, 0.071 | 0.211 | 0.017 | -0.026, 0.060 | 0.440 |
| MFIS | 0.054 | 0.008, 0.101 | 0.022 | 0.032 | -0.014, 0.077 | 0.169 |
| MSSS | -0.018 | -0.063, 0.026 | 0.414 | -0.015 | -0.060, 0.030 | 0.508 |
| SF-36 Physical QOL | -0.083 | -0.127, -0.039 | <0.001 | -0.056 | -0.102, -0.010 | 0.016 |
| SF-36 Mental QOL | -0.017 | -0.060, 0.025 | 0.419 | 0.001 | -0.040, 0.042 | 0.955 |
| Legend: CES-D = Center for Epidemiology Scale for Depression; MFIS = Modified Fatigue Impact Scale; MSSS = Modified Social Support Survey; QOL = quality of life; BMI = body mass index; DMT = disease modifying therapy. Mixed linear regression models use data from the last patient visit only, with multivariable model including the following covariates: age, gender, smoking history, and DMT use. Continuous variables are Z-transformed such that one unit increase represents one standard deviation change. | | | | | | |

| Table S2: Predictors of natural log-transformed MLR in univariate analysis (last subject visit) | | | | | | |
| --- | --- | --- | --- | --- | --- | --- |
| Variable | *Univariable* | | | *Multivariable* | | |
|  | *β* | 95% CI | *p* | *β* | 95% CI | *p* |
| Age | 0.052 | 0.013, 0.092 | 0.009 | 0.054 | 0.017, 0.092 | 0.005 |
| Disease Duration | 0.054 | 0.016, 0.091 | 0.006 | 0.025 | -0.016, 0.066 | 0.238 |
| Gender |  |  |  |  |  |  |
| - Female | - | - | - | - | - | - |
| - Male | 0.209 | 0.120, 0.298 | <0.001 | 0.208 | 0.122, 0.293 | <0.001 |
| Smoking History |  |  |  |  |  |  |
| - No/Unknown | - | - | - | - | - | - |
| - Yes | 0.001 | -0.087, 0.089 | 0.986 | 0.009 | -0.068, 0.087 | 0.810 |
| BMI | -0.027 | -0.070, 0.016 | 0.214 | -0.028 | -0.069, 0.013 | 0.187 |
| DMT |  |  |  |  |  |  |
| - Untreated | - | - | - | - | - | - |
| - Interferon-β | 0.067 | -0.028, 0.161 | 0.166 | 0.091 | -0.008, 0.190 | 0.073 |
| - Cyclophosphamide | 0.445 | 0.216, 0.674 | <0.001 | 0.457 | 0.216, 0.698 | <0.001 |
| - Fingolimod | 1.208 | 0.913, 1.504 | <0.001 | 1.225 | 0.916, 1.533 | <0.001 |
| - Glatiramer Acetate | -0.109 | -0.246, 0.028 | 0.119 | -0.111 | -0.251, 0.028 | 0.117 |
| - Mycophenolate | 0.012 | -0.149, 0.172 | 0.887 | 0.007 | -0.154, 0.168 | 0.936 |
| - Natalizumab | -0.343 | -0.496, -0.190 | <0.001 | -0.351 | -0.504, -0.198 | <0.001 |
| - Other | 0.171 | -0.058, 0.400 | 0.143 | 0.124 | -0.108, 0.356 | 0.294 |
| CES-D | -0.005 | -0.045, 0.035 | 0.796 | -0.002 | -0.040, 0.036 | 0.926 |
| MFIS | 0.002 | -0.040, 0.045 | 0.916 | 0.005 | -0.035, 0.045 | 0.815 |
| MSSS | 0.017 | -0.024, 0.058 | 0.413 | 0.023 | -0.017, 0.062 | 0.264 |
| SF-36 Physical QOL | -0.031 | -0.072, 0.011 | 0.144 | -0.034 | -0.075, 0.006 | 0.098 |
| SF-36 Mental QOL | 0.007 | -0.032, 0.046 | 0.737 | 0.018 | -0.019, 0.054 | 0.341 |
| Legend: CES-D = Center for Epidemiology Scale for Depression; MFIS = Modified Fatigue Impact Scale; MSSS = Modified Social Support Survey; QOL = quality of life. Mixed linear regression models use data from the last patient visit only, with multivariable model including the following covariates: age, gender, smoking history, and DMT use. Continuous variables are Z-transformed such that one unit increase represents one standard deviation change. | | | | | | |

| Table S3: Natural log-transformed NLR as a predictor of objective MS disease outcomes using last subject visit only | | | | | | | | | |
| --- | --- | --- | --- | --- | --- | --- | --- | --- | --- |
| Outcome | *Univariable* | | | *Multivariable Model 1* | | | *Multivariable Model 2* | | |
|  | *β* | 95% CI | *p* | *β* | 95% CI | *p* | *β* | 95% CI | *p* |
| EDSS | 1.024 | 0.691, 1.358 | <0.001 | 0.672 | 0.324, 1.019 | <0.001 | 0.309 | 0.036, 0.583 | 0.027 |
| RRMS v PMS | 1.162 | 0.690, 1.635 | <0.001 | 0.882 | 0.279, 1.484 | 0.004 | 0.895 | 0.164, 1.626 | 0.016 |
| BPF | -0.018 | -0.028, -0.008 | 0.001 | -0.010 | -0.02, 0.000 | 0.048 | -0.008 | -0.018, 0.002 | 0.137 |
| T2LV | 0.137 | 0.010, 0.265 | 0.035 | 0.082 | -0.055, 0.219 | 0.241 | 0.076 | -0.066, 0.218 | 0.294 |
| Legend: EDSS = Expanded Disability Status Scale; RRMS = relapsing-remitting multiple sclerosis; PMS = progressive multiple sclerosis; BPF = brain parenchymal fraction; T2LV = cerebral T2-hyperintense lesion volume. All statistical models are linear mixed effects regressions. Multivariable model #1 includes the following covariates: age, disease duration, gender, smoking history, and specific DMT use. Model #2 includes the covariates: age, disease duration, gender, smoking history, specific DMT use, CES-D score, MSSS score, SF-36 physical and mental composite scores. | | | | | | | | | |

| Table S4: Natural log-transformed MLR as a predictor of objective MS disease outcomes using last subject visit only | | | | | | | | | |
| --- | --- | --- | --- | --- | --- | --- | --- | --- | --- |
| Outcome | *Univariable* | | | *Multivariable Model 1* | | | *Multivariable Model 2* | | |
|  | *β* | 95% CI | *p* | *β* | 95% CI | *p* | *β* | 95% CI | *p* |
| EDSS | 0.776 | 0.407, 1.144 | <0.001 | 0.697 | 0.304, 1.091 | 0.001 | 0.332 | 0.025, 0.639 | 0.034 |
| RRMS v PMS | 0.962 | 0.486, 1.439 | <0.001 | 0.914 | 0.270, 1.559 | 0.005 | 0.597 | -0.278, 1.472 | 0.181 |
| BPF | -0.019 | -0.029, -0.008 | 0.001 | -0.008 | -0.019, 0.003 | 0.143 | -0.004 | -0.015, 0.007 | 0.462 |
| T2LV | 0.131 | 0.000, 0.262 | 0.050 | 0.083 | -0.062, 0.229 | 0.261 | 0.086 | -0.064, 0.237 | 0.260 |
| Legend: EDSS = Expanded Disability Status Scale; RRMS = relapsing-remitting multiple sclerosis; PMS = progressive multiple sclerosis; BPF = brain parenchymal fraction; T2LV = cerebral T2-hyperintense lesion volume. All statistical models are linear mixed effects regressions. Multivariable model #1 includes the following covariates: age, disease duration, gender, smoking history, and specific DMT use. Model #2 includes the covariates: age, disease duration, gender, smoking history, specific DMT use, CES-D score, MSSS score, SF-36 physical and mental composite scores. | | | | | | | | | |

Sensitivity analysis using untreated patients only (N=146)

| Table S5: Log-transformed NLR as a predictor of objective MS disease outcomes using untreated subjects | | | | | | | | | |
| --- | --- | --- | --- | --- | --- | --- | --- | --- | --- |
| Outcome | *Univariable* | | | *Multivariable Model 1* | | | *Multivariable Model 2* | | |
|  | *β* | 95% CI | *p* | *β* | 95% CI | *p* | *β* | 95% CI | *p* |
| EDSS | 0.657 | 0.182, 1.131 | 0.007 | 0.721 | 0.283, 1.159 | 0.001 | 0.623 | 0.180, 1.067 | 0.006 |
| RRMS v PMS | 1.015 | 0.231, 1.800 | 0.011 | 1.868 | 1.048, 2.688 | <0.001 | 1.457 | 0.509, 2.405 | 0.003 |
| BPF | -0.001 | -0.010, 0.007 | 0.789 | -0.004 | -0.011, 0.003 | 0.299 | -0.003 | -0.010, 0.004 | 0.405 |
| T2LV | 0.009 | -0.075, 0.094 | 0.834 | 0.028 | -0.058, 0.114 | 0.530 | 0.022 | -0.069, 0.113 | 0.633 |
| Legend: EDSS = Expanded Disability Status Scale; RRMS = relapsing-remitting multiple sclerosis; PMS = progressive multiple sclerosis; BPF = brain parenchymal fraction; T2LV = cerebral T2-hyperintense lesion volume. All statistical models are linear mixed effects regressions. Multivariable model #1 includes the following covariates: age, disease duration, gender, smoking history, and specific DMT use. Model #2 includes the covariates: age, disease duration, gender, smoking history, specific DMT use, CES-D score, MSSS score, SF-36 physical and mental composite scores. | | | | | | | | | |

| Table S6: Log-transformed MLR as a predictor of objective MS disease outcomes using untreated subjects | | | | | | | | | |
| --- | --- | --- | --- | --- | --- | --- | --- | --- | --- |
| Outcome | *Univariable* | | | *Multivariable Model 1* | | | *Multivariable Model 2* | | |
|  | *β* | 95% CI | *p* | *β* | 95% CI | *p* | *β* | 95% CI | *p* |
| EDSS | 1.133 | 0.599, 1.667 | <0.001 | 0.870 | 0.340, 1.400 | 0.001 | 0.712 | 0.171, 1.253 | 0.010 |
| RRMS v PMS | 1.423 | 0.290, 2.556 | 0.014 | 2.062 | 0.996, 3.128 | <0.001 | 1.617 | 0.394, 2.840 | 0.010 |
| BPF | -0.010 | -0.019, -0.002 | 0.020 | -0.006 | -0.013, 0.002 | 0.149 | -0.007 | -0.014, 0.001 | 0.069 |
| T2LV | -0.008 | -0.097, 0.081 | 0.863 | -0.022 | -0.113, 0.069 | 0.641 | -0.059 | -0.159, 0.041 | 0.244 |
| Legend: EDSS = Expanded Disability Status Scale; RRMS = relapsing-remitting multiple sclerosis; PMS = progressive multiple sclerosis; BPF = brain parenchymal fraction; T2LV = cerebral T2-hyperintense lesion volume. All statistical models are linear mixed effects regressions. Multivariable model #1 includes the following covariates: age, disease duration, gender, smoking history, and specific DMT use. Model #2 includes the covariates: age, disease duration, gender, smoking history, specific DMT use, CES-D score, MSSS score, SF-36 physical and mental composite scores. | | | | | | | | | |
